# Rapid and high-sensitivity quantitative fluorescent immunoassay for detection of *Klebsiella pneumoniae* carbapenemase

**DOI:** 10.3389/fmicb.2026.1806227

**Published:** 2026-04-29

**Authors:** Menglu Mao, Keke Li, Chenyao Lin, Xuedan Qiu, Zhen Zhang, Dumei Ma, Qingcao Li

**Affiliations:** 1 Department of Clinical Laboratory, The Affiliated Lihuili Hospital of Ningbo University, Ningbo, China; 2 Genobio Pharmaceutical Co., Ltd., Tianjin, China; 3 School of Laboratory Medicine and Bioengineering, Hangzhou Medical College, Hangzhou, China; 4 Key Laboratory of Advanced Mass Spectrometry and Molecular Analysis of Zhejiang Province, School of Materials Science and Chemical Engineering, Institute of Mass Spectrometry, Ningbo University, Ningbo, China; 5 State Key Laboratory for Diagnosis and Treatment of Severe Zoonostic Infectious Disease, Wuhan, China

**Keywords:** fluorescent immunoassay, fluoroimmunoassay, *Klebsiella pneumoniae* carbapenemase, quantification, quantitative detection, rapid detection

## Abstract

**Introduction:**

Early and precise identification of *Klebsiella pneumoniae* carbapenemase (KPC) is critical for guiding effective treatment and curbing the spread of carbapenem-resistant pathogens. The mainstream detection methods targeting KPC are exclusively qualitative, lacking reliable quantitative approaches. To address the need for a rapid and quantitative method, this study developed a sensitive fluorescence immunoassay.

**Methods:**

The assay employs a sandwich immunoassay format, which utilizes carboxylated magnetic agarose microspheres for capture and time-resolved fluorescent microspheres for detection. It enables quantification of the KPC protein within approximately 1 h. The analytical performance of the assay was systematically evaluated, including its sensitivity, linear range, specificity and accuracy.

**Results:**

Under optimal experimental conditions, it exhibits a wide linear range (0.25–1000 ng/mL) and a low detection limit of 0.194 ng/mL, and shows no cross-reactivity with other common carbapenemases. Recovery rates in bacterial lysates ranged from 96.7 to 108.4%. Compared to a commercial immunochromatographic test, this method demonstrated superior sensitivity.

**Discussion:**

The primary advantage of this fluorescent immunoassay is its ability to achieve rapid, sensitive and quantitative detection of KPC, which addresses a key limitation of current qualitative methods. Its wide linear range and low detection limit enable precise monitoring of KPC expression level variations, offering a novel tool for the clinical assessment of bacterial resistance burden.

## 1 Introduction

Antimicrobial resistance (AMR) has developed into a crucial challenge for public health worldwide (Shafiq et al., 2021; Murray et al., 2022; Wozniak et al., 2022). With the growing prevalence of multidrug-resistant (MDR) bacteria, carbapenems serve as a final option for combating severe infections (Tamma et al., 2023; Mishra et al., 2025). The infection rate of carbapenem-resistant Gram-negative bacilli (CRGNB) has risen considerably due to the widespread use of carbapenems, complicating clinical treatment and leading to higher rates of morbidity and mortality (Marques et al., 2015; Naghavi et al., 2024). The World Health Organization (WHO) has classified carbapenem-resistant *Enterobacterales* (CRE) and carbapenem-resistant *Pseudomonas aeruginosa* (CRPA) as critical and high-priority pathogens, respectively (Jesudason, 2024).

The production of carbapenemases represents a primary resistance mechanism in CRGNB (Hansen, 2021). Bacterial resistance profiles and treatment regimens vary significantly according to the specific type of produced carbapenemase (Tamma and Hsu, 2019; Tompkins and van Duin, 2021; Jean et al., 2022; Lu et al., 2022). The critical importance of carbapenemase typing has been underscored by recent updates from both standards-setting and professional organizations. The Clinical and Laboratory Standards Institute (CLSI) now recommends carbapenemase testing for most *Enterobacterales* resistant to at least one carbapenem, emphasizing differentiation of key enzymes such as KPC, New Delhi metallo-β-lactamase (NDM), and OXA-48-like (oxacillinase-48-like) (Simner, 2026). The guidelines issued by the Infectious Diseases Society of America (IDSA) also explicitly encourage that clinical laboratories perform carbapenemase testing to guide treatment decisions (Tamma et al., 2024). Studies have demonstrated that timely carbapenemase typing is critical for patient outcomes. In patients with KPC-producing *Klebsiella pneumoniae* bloodstream infections, each 24-h delay in appropriate therapy increases 30-day mortality risk (hazard ratio 1.38). Mortality rates are 29.1% for those treated within 24 h versus 63.8% for later treatment (Falcone et al., 2020). Rapid diagnostic methods have been shown to reduce time to active therapy from 50 to 24 h and lower 30-day mortality from 47 to 24% (Satlin et al., 2022). Therefore, optimal patient management depends on combining rapid and accurate enzyme typing with a thorough assessment of the patient’s clinical condition, the local epidemiology of resistant bacteria, and the most current treatment guidelines. In this context, the clinical microbiology laboratory serves a crucial role in ensuring the judicious use of antibiotics and strengthening infection prevention.

A variety of methods for detecting carbapenemases have been developed, each with distinct advantages and limitations (Caliskan-Aydogan and Alocilja, 2023; Simner et al., 2024). Molecular detection techniques, such as polymerase chain reaction (PCR), gene chips, and next-generation sequencing (NGS), offer excellent discriminatory power but are relatively complex to operate, with high costs and specialized equipment limiting their widespread use. The modified carbapenem inactivation method (mCIM) exhibits high specificity and sensitivity with low cost, but has a prolonged turnaround time and limited enzyme typing capability (Tsai et al., 2020; Zhong et al., 2020). The Carba NP test is relatively rapid yet involves complex operations, requires specialized reagents and shows low sensitivity to certain enzyme types (Österblad et al., 2014; Maccari et al., 2024). Matrix-assisted laser desorption/ionization time-of-flight mass spectrometry (MALDI-TOF MS) requires expensive equipment and sophisticated data analysis software, making it inaccessible to some laboratories (Gato et al., 2022). Immunochromatographic tests are rapid, user-friendly, and suitable for routine laboratories, yet they only offer qualitative or semi-quantitative results, and their sensitivity may be insufficient for low-burden samples (Josa et al., 2022). Moreover, existing techniques are confined to specific scenarios by their inherent limitations. The mainstream detection methods targeting specific enzyme types are exclusively qualitative, lacking reliable quantitative approaches. Therefore, there is an urgent need to establish a rapid and accurate method for carbapenemase detection and typing.

Among the various carbapenemases, KPC was selected as the initial target for this study for two main reasons. First, KPC remains one of the most widely distributed carbapenemases globally, dominating the epidemiology of CRE in the United States, Europe (particularly Italy and Greece), Latin America, and Asia, including China (Satlin et al., 2022; Cai et al., 2024). Data from the SMART global surveillance program between 2008 and 2014 showed that among the collected carbapenemase-producing *Enterobacterales*, KPC-positive isolates were the most common (Thomson and Munson, 2017). This widespread distribution is largely attributed to its high transmissibility. Its coding gene (*bla*_KPC_) is commonly located on mobile genetic elements (MGE) like transposon Tn*4401*, and is highly transmissible horizontally among different strains and species (David et al., 2020; Tang et al., 2021). Second, the platform established for KPC detection can serve as a template that is readily adaptable to other clinically relevant carbapenemases, such as NDM, OXA-48, Verona integron-encoded metallo-β-lactamase (VIM), and imipenem-hydrolyzing metallo-β-lactamase (IMP).

Herein, we proposed a highly sensitive fluorescence immunoassay utilizing magnetic microparticles and fluorescently labeled antibodies for the absolute quantification of KPC in clinical specimens. First, the KPC protein was specifically captured using carboxylated magnetic agarose microspheres immobilized with capture antibodies, achieving efficient enrichment of KPC while reducing the required sample volume and processing time. Subsequently, detection antibodies conjugated with time-resolved fluorescent microspheres were added to form a dual-antibody sandwich complex. Owing to the low background and high loading capacity of these microspheres, the assay enables highly sensitive fluorescence signal detection. With its low detection limit and wide linear range, this method is expected to enhance the detection capability of KPC in cases of low bacterial load or expression. It may facilitate a more precise assessment of drug resistance levels, and provide a novel KPC detection strategy for clinical practice that is rapid, sensitive, quantitative, and readily applicable.

## 2 Materials and methods

### 2.1 Materials and bacterial strains

In this study, the recombinant KPC, VIM, IMP, NDM, and OXA-48 proteins, anti-KPC monoclonal antibodies (mAb1 and mAb2), and bacterial lysis buffer were supplied by Genobio Pharmaceutical Co., Ltd. (Tianjin, China). Time-resolved fluorescent microspheres were purchased from Bangs Laboratories, Inc. (Fishers, IN, United States). The following chemicals were obtained from Macklin Inc. (Shanghai, China): ferric chloride hexahydrate (FeCl_3_⋅6H_2_O), sodium acetate, sodium citrate solution (Na_3_C_6_H_5_O_7_⋅2H_2_O), liquid paraffin, petroleum ether, sodium hydroxide (NaOH), Tween 20, and MES buffer. Sodium borohydride (NaBH_4_) and sodium carbonate (Na_2_CO_3_) were sourced from Aladdin Scientific Inc. (Shanghai, China). N-(3-Dimethylaminopropyl)-N′-ethylcarbodiimide (EDC) and N-hydroxysuccinimide (NHS) were purchased from Merck KGaA (Darmstadt, Germany). Additionally, agarose, Span 80, epichlorohydrin, dimethyl sulfoxide (DMSO), iminodiacetic acid (IDA), PBS buffer, bovine serum albumin (BSA), ethanol, and ethylene glycol were acquired from Sangon Biotech Co., Ltd. (Shanghai, China). All isolated strains were obtained from The Affiliated Lihuili Hospital of Ningbo University, and the study received approval from the Ethics Committee of The Affiliated Lihuili Hospital of Ningbo University (KY2023SL041-01).

### 2.2 Synthesis of Fe_3_O_4_@agarose-mAb1

Fe_3_O_4_ nanoparticles were synthesized using a method that has been previously documented (Zheng et al., 2015). The synthesis of Fe_3_O_4_@agarose-IDA was performed based on previous literature with appropriate modifications (Zhao et al., 2023), and the details are provided in Supplementary material. Next, 5 mg of Fe_3_O_4_@agarose-IDA microspheres were sequentially washed three times with ethanol, deionized water, and MES buffer (pH 6.0). Fe_3_O_4_@agarose-IDA was then dispersed in 1 mL of MES buffer (pH 6.0) with 10 mg EDC and 10 mg NHS, and shaken at 75 rpm for 15 min at room temperature. After rapid washing with MES buffer (pH 6.0), the activated microspheres were incubated with 500 μL of anti-KPC mAb1 (1 mg/mL in PBS, pH 7.4) and rotated at 45 rpm for 3 h at room temperature. Finally, the microspheres were incubated with 1 mL of blocking buffer (1% BSA, 0.05% Tween-20 in PBS, pH 7.4) for 1.5 h, washed three times with PBS (pH 7.4), resuspended in 1 mL of PBS (pH 7.4), and stored at 4 °C for further use.

### 2.3 Conjugation of fluorescent microspheres with mAb2

First, a volume of 22 μL from the time-resolved fluorescent microspheres suspension was pipetted into 400 μL of MES buffer (pH 6.0) and vortexed thoroughly. The mixture was centrifuged at 11580 × *g* for 15 min, washed twice, and resuspended in 400 μL of MES buffer (pH 6.0). The resuspended microspheres were sonicated on ice using a probe sonicator at 20% power for 3 min. Subsequently, 40 μg of NHS and 40 μg of EDC were added to the microsphere suspension. The mixture was then vortexed, sonicated in an ice bath for 3 min, and activated by shaking at 75 rpm for 30 min at room temperature in the dark. The activated microspheres were then collected by centrifugation at 11580 × *g* for 15 min. The supernatant was discarded, and the pellet was resuspended in 400 μL of MES buffer (pH 7.4) and sonicated for 3 min. After this, 100 μL of anti-KPC mAb2 (1 mg/mL) was added to the activated microspheres, followed by sonication on ice for 3 min. The mixture was then incubated with shaking at 50 rpm for 2 h at room temperature in the dark. After incubation, the mixture was centrifuged at 11,580 × *g* for 15 min, and the supernatant was discarded. The microspheres were then washed with 400 μL of MES buffer (pH 7.4) and resuspended by sonication. Next, 250 μL of blocking solution (2% BSA in MES, pH 7.4) was added and the mixture was incubated with shaking at 50 rpm for 1 h at room temperature in the dark. The mixture was centrifuged at 11,580 × *g* for 15 min, and the supernatant was removed. The microspheres were then resuspended in 400 μL of MES buffer (pH 7.4) followed by 3 min of sonication. The microspheres were centrifuged again at 11,580 × *g* for 15 min, resuspended in 200 μL of PBST (0.05% Tween-20 in PBS, pH 7.4), and subjected to sonication on ice for 3 min. The resulting suspension (mAb2-conjugated fluorescent microspheres) was stored at 4 °C in the dark until further use.

### 2.4 KPC detection procedure

A mixture of 100 μL of Fe_3_O_4_@agarose-mAb1 and 100 μL of the sample was incubated at 37 °C with shaking at 1250 rpm for 30 min. The precipitate was fixed under magnetic force and rinsed three times with PBS (pH 7.4). After washing, the magnetic beads were then resuspended in 100 μL of PBS (pH 7.4). Then, a volume of 100 μL of mAb2-conjugated fluorescent microspheres was introduced and incubated at 37°C with shaking at 1250 rpm for 30 minunder light-protected conditions. Following magnetic separation, the precipitate was collected and washed three times with PBS (pH 7.4). The product was dispersed in 200 μL of PBS (pH 7.4). Fluorescence was then measured on an RF-6000 spectrofluorophotometer (Shimadzu, Kyoto, Japan). The excitation and emission wavelengths were set at 344 nm and 614 nm, respectively, with the fluorescence signal recorded in the range of 550–650 nm. All fluorescence detections in this study were performed using the above parameters.

### 2.5 Analytical performance evaluation

KPC protein was serially diluted with PBS (pH 7.4) to concentrations of 5000, 1000, 500, 100, 50, 10, 5, 1, 0.5, 0.25, and 0.125 ng/mL. Under optimal assay conditions, these diluted standards were analyzed using the proposed method, and the corresponding fluorescence signals were recorded for each KPC concentration.

The specificity of the assay was first evaluated by assessing potential cross-reactivity between KPC and other common carbapenemases (VIM, IMP, NDM, OXA-48). These proteins were assayed at 25 ng/mL under the same experimental conditions, with PBS serving as the negative control. Then, to evaluate potential interference from other β-lactamases in detection, clinically isolated strains previously characterized via PCR were selected for validation testing. Assessed strains included KPC-producing isolates, as well as strains negative for *bla*_KPC_ but carrying genes encoding other β-lactamases. The test included the following β-lactamases: DHA, CTX-M-1, CTX-M-3, CTX-M-9, CTX-M-14, and SHV. Detailed information on the tested strains is listed in Supplementary Table 1. All isolates were cultured on blood agar plates (Autobio, Zhengzhou) at 37°C for 16–18 h. Single colonies were picked and suspended in sterile saline, then adjusted to 0.5 McFarland using a turbidimeter. These prepared bacterial suspensions were then analyzed by the proposed method under optimal experimental conditions.

Recovery rates were subsequently determined by spiking KPC protein at various concentrations into bacterial lysates, in order to evaluate the accuracy and practical applicability of the proposed method. Standard strains of *Klebsiella pneumoniae* ATCC BAA-1706 and *Pseudomonas aeruginosa* ATCC 27853, both non-KPC producers, were employed to prepare the tested matrix in this experiment. The standard strains were cultured on blood agar plates at 37°C for 16–18 h. Single colonies were then picked and resuspended in 5 drops of bacterial lysis buffer and thoroughly mixed by vortexing. A 100 μL aliquot of the lysate was spiked with an appropriate amount of KPC protein, with three replicate samples prepared for each concentration. These samples were subsequently analyzed using this method.

### 2.6 Method comparison

To evaluate the feasibility of the proposed method in real samples, this assay was compared separately with PCR and the commercial Colloidal Gold Immunochromatographic Assay (CGIA) method.

#### 2.6.1 Comparison with PCR

A total of 31 clinical isolates were selected for evaluation, including *Klebsiella pneumoniae*, *Pseudomonas aeruginosa*, *Escherichia coli*, and *Acinetobacter baumannii*. Detailed information on the strains is provided in Supplementary Table 2. The results obtained by the proposed method were compared with those from PCR, and the Kappa test was used to analyze the consistency between the two approaches. The experimental procedures of the proposed method were performed as follows. All isolates were cultured on blood agar plates at 37°C for 16–18 h. Single colonies were picked and suspended in sterile saline, followed by adjustment to a 0.5 McFarland turbidity standard using a turbidimeter. The prepared bacterial suspensions were subsequently detected by the proposed method under optimal experimental conditions.

#### 2.6.2 Comparison with CGIA

The KPC-producing standard strain *Klebsiella pneumoniae* ATCC BAA-1705 was streaked onto a blood agar plate and incubated at 37°C for 16–18 h. A single colony was selected and resuspended in sterile saline to prepare a bacterial suspension adjusted to 0.5 McFarland standard (∼10^8^ CFU/mL). Serial 10-fold dilutions were then performed to obtain concentrations ranging from ∼10^7^ to ∼10^3^ CFU/mL. For each concentration level, three replicate samples were prepared, without any exogenous KPC added. Subsequently, 200 μL of each diluted bacterial suspension and a PBS negative control were mixed with 5 drops of bacterial lysis buffer by vortexing. Afterward, 100 μL of each lysate was used for detection with the proposed method, and 50 μL was applied to the colloidal gold test strip. The colloidal gold test results were read at 10 min.

### 2.7 Statistical analysis

Statistical analysis was conducted with Origin 2022 (OriginLab, Northampton, Massachusetts, United States). The results are expressed as mean ± standard deviation (SD) from replicate experiments. One-way analysis of variance (ANOVA) followed by Tukey’s *post-hoc* test was used to analyze intergroup differences during parameter optimization and specificity verification, with the significance level set at *p* < 0.05. The consistency between two methods was analyzed using the Kappa test.

## 3 Results

### 3.1 Assay workflow and characterization of key components

**FIGURE 1:**
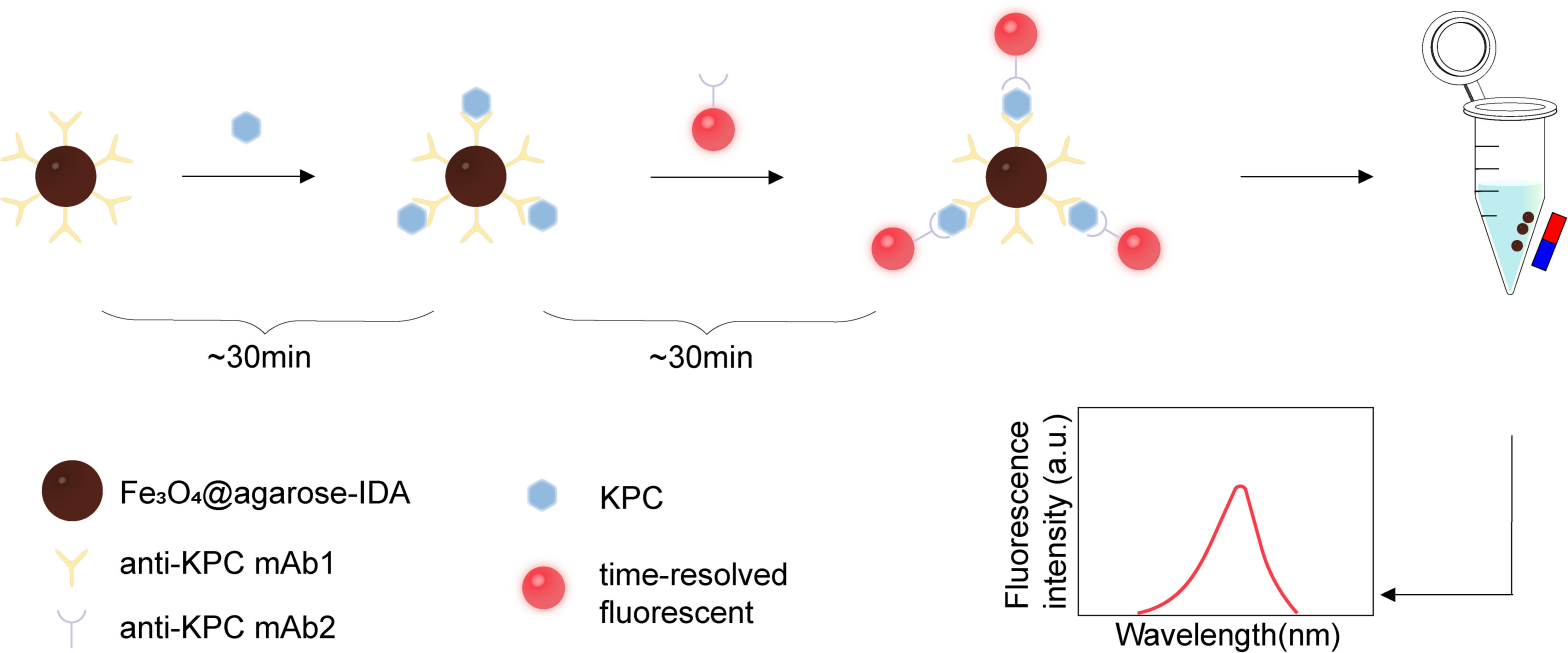
Schematic illustration of the fluorescent immunoassay for KPC detection.

In this detection strategy, KPC protein in the sample is initially captured by magnetic microspheres conjugated with anti-KPC monoclonal antibody 1 (anti-KPC mAb1). The captured protein then binds to a fluorescent probe labeled with anti-KPC monoclonal antibody 2 (anti-KPC mAb2), forming a sandwich immunocomplex for subsequent fluorescence detection. As illustrated in Figure 1, carboxylated magnetic microspheres (Fe_3_O_4_@agarose-IDA) serve as a solid-phase support and are conjugated with anti-KPC mAb1 to prepare immunomagnetic capture carriers. Following the addition of KPC protein or test samples, the target analyte is captured and enriched by these immunomagnetic carriers. Following a 30-min incubation, the mixture is magnetically separated, and time-resolved fluorescent microspheres conjugated with anti-KPC mAb2 are added to form a double-antibody sandwich complex. After another 30 min of reaction, the resulting complex is collected magnetically and resuspended in 200 μL of PBS. The emission intensity of the final product was detected at 614 nm, using an excitation wavelength of 344 nm. Quantitative analysis is performed based on the correlation between the fluorescence intensity and the KPC protein concentration.

**FIGURE 2:**
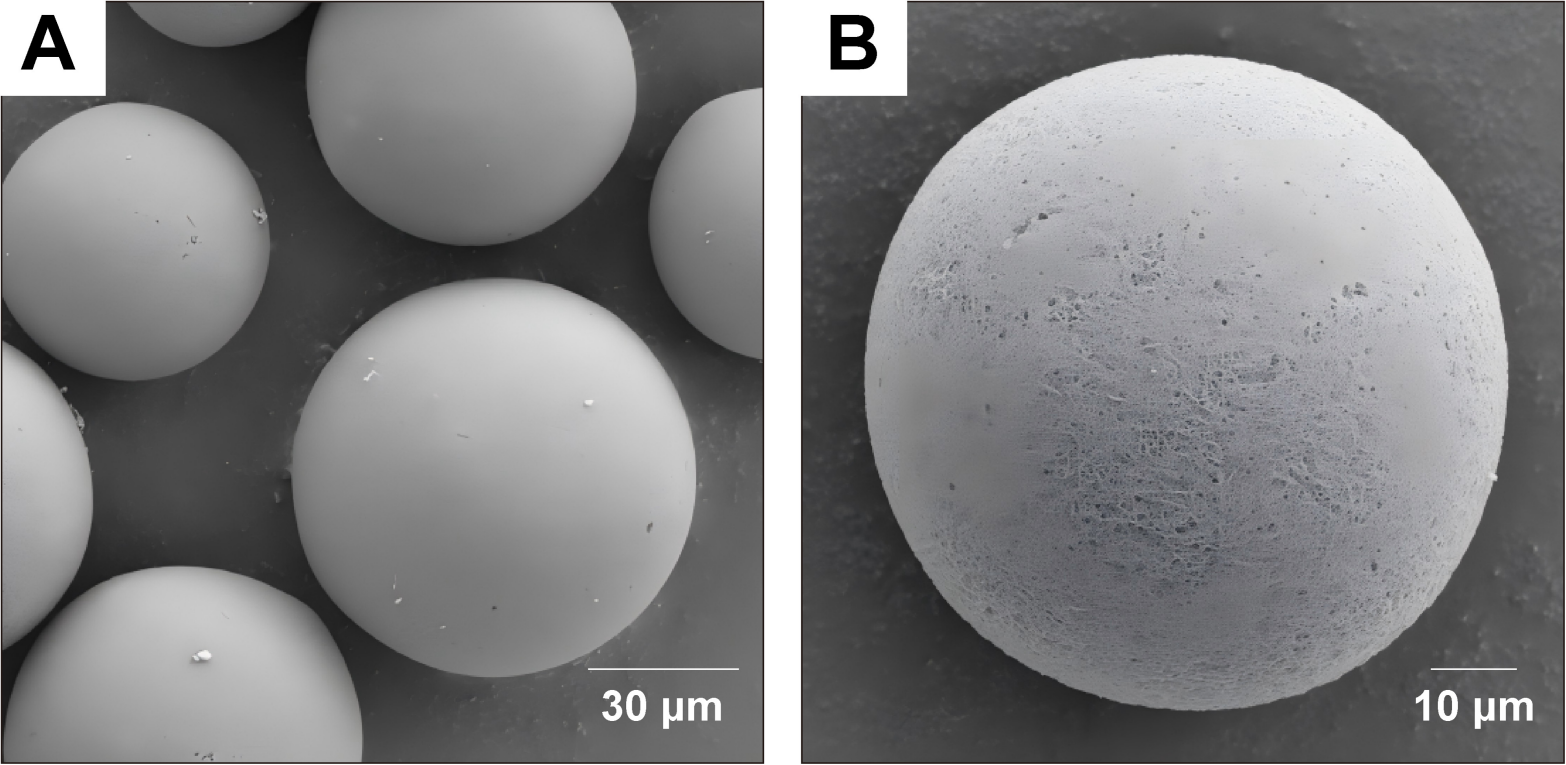
SEM images of Fe_3_O_4_@agarose-IDA**. (A)** Scale bar: 30 μm, magnification: 4,000 × . **(B)** Scale bar: 10 μm, magnification: 8,000 × .

**FIGURE 3:**
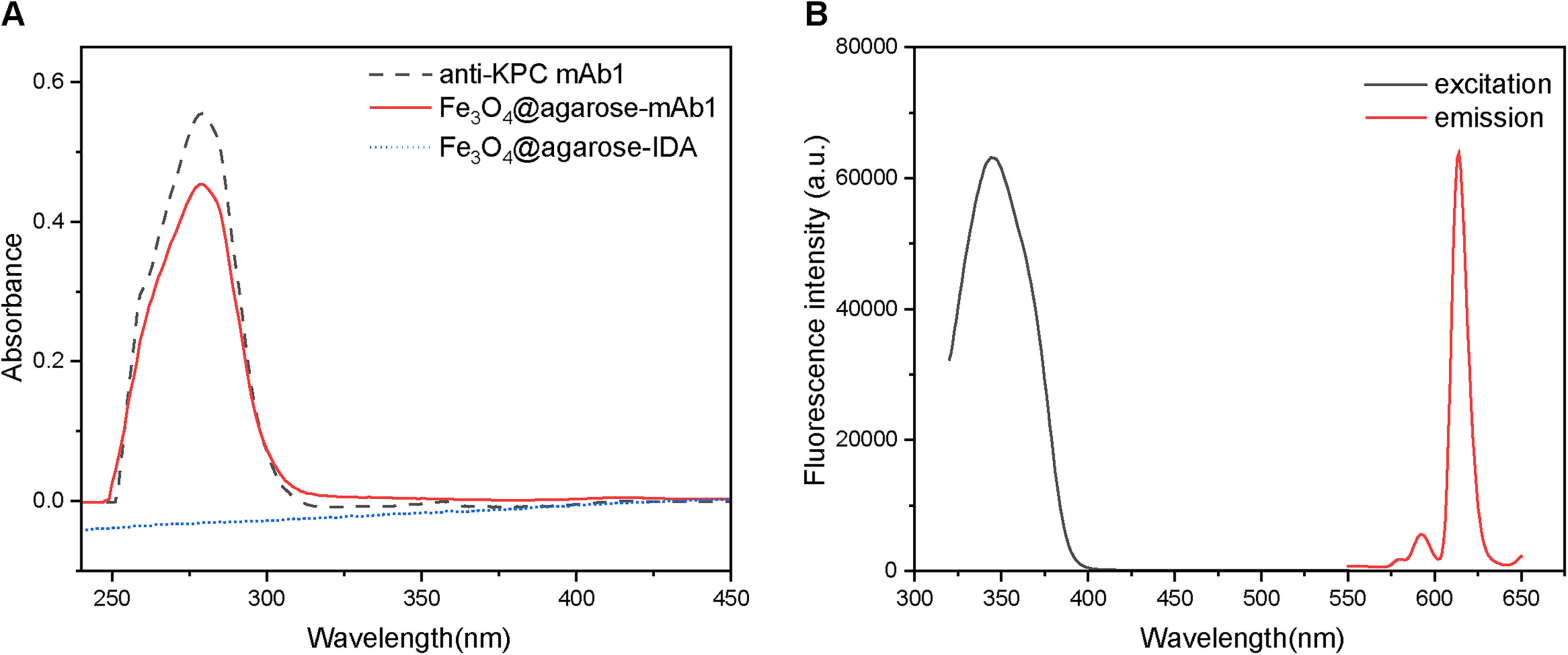
**(A)** UV-Vis absorption spectra of anti-KPC mAb1, Fe_3_O_4_@agarose-mAb1, and Fe_3_O_4_@agarose-IDA (top to bottom). **(B)** Excitation and emission spectra of mAb2-conjugated fluorescent microspheres.

To confirm the successful synthesis of the experimental materials, the following characterizations were performed. The scanning electron microscopy (SEM) images of Fe_3_O_4_@agarose-IDA are shown in Figures 2A,B. The Fe_3_O_4_@agarose-IDA microspheres were highly monodisperse and exhibited a spherical structure, measuring approximately 50–100 μm. Ultraviolet-visible (UV-Vis) absorption spectra of anti-KPC mAb1, Fe_3_O_4_@agarose-IDA, and Fe_3_O_4_@agarose-mAb1 were measured separately. It is commonly known that monoclonal antibodies exhibit UV absorption at a wavelength of 280 nm (Mochizuki et al., 2025). As illustrated in Figure 3A, anti-KPC mAb1 exhibited a characteristic absorption peak at 280 nm, while Fe_3_O_4_@agarose-IDA showed no obvious absorption peak. And Fe_3_O_4_@agarose-mAb1 displayed an absorption peak at 280 nm, indicating the successful conjugation of anti-KPC mAb1 to the magnetic microspheres. The excitation and emission spectra of the mAb2-conjugated fluorescent microspheres are presented in Figure 3B, exhibiting a maximum excitation wavelength at 344 nm and a maximum emission wavelength at 614 nm.

### 3.2 Optimization of experimental parameters

**FIGURE 4:**
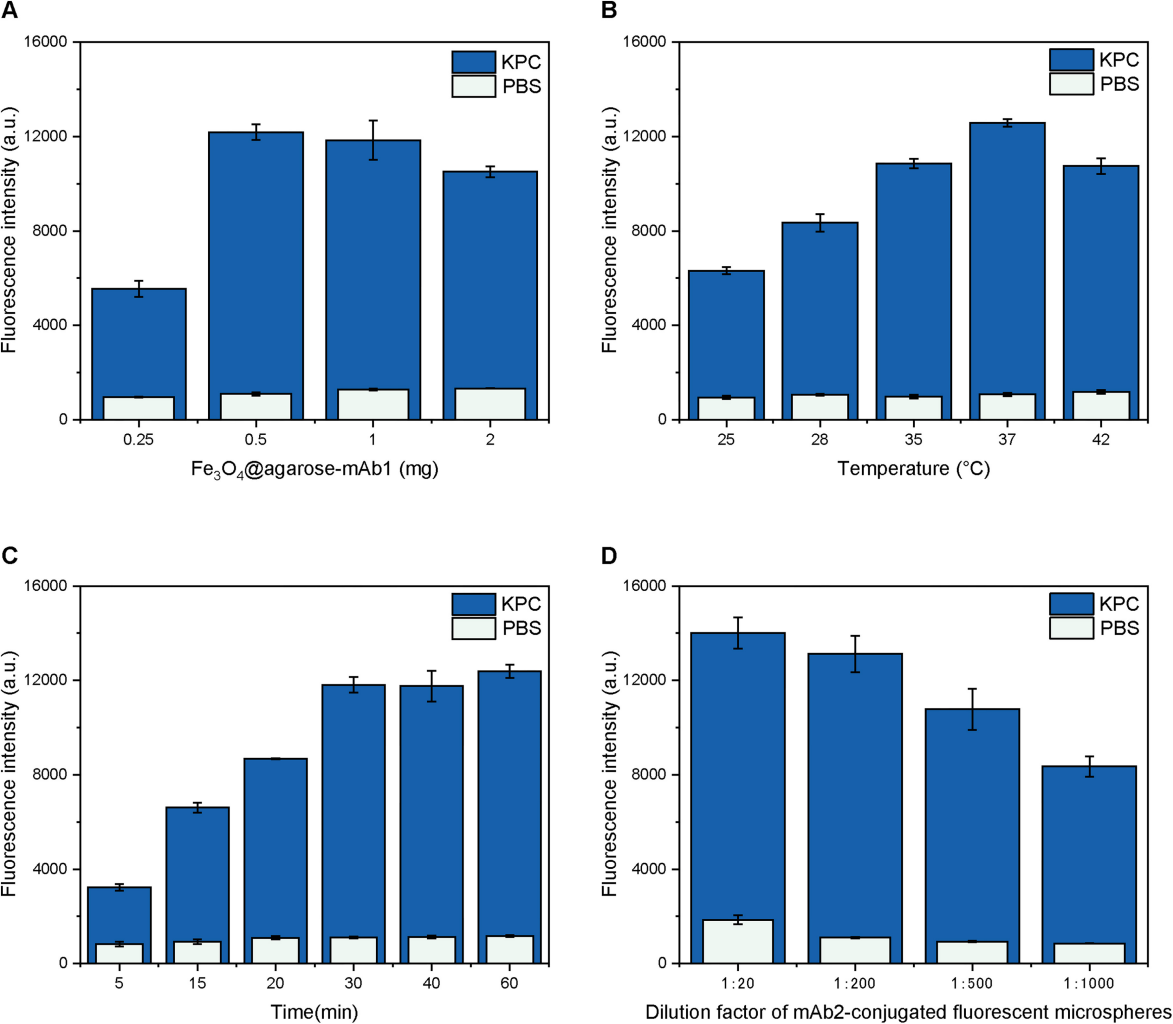
Fluorescence intensity under varying conditions of **(A)** magnetic microspheres dosage, **(B)** reaction temperature, **(C)** incubation time, and **(D)** dilution ratio of mAb2-conjugated fluorescent microspheres. Dark bars represent total fluorescence intensity, while light bars denote fluorescence intensity of PBS negative controls. Error bars indicate the standard deviation (SD) of the mean calculated from three replicates.

**FIGURE 5:**
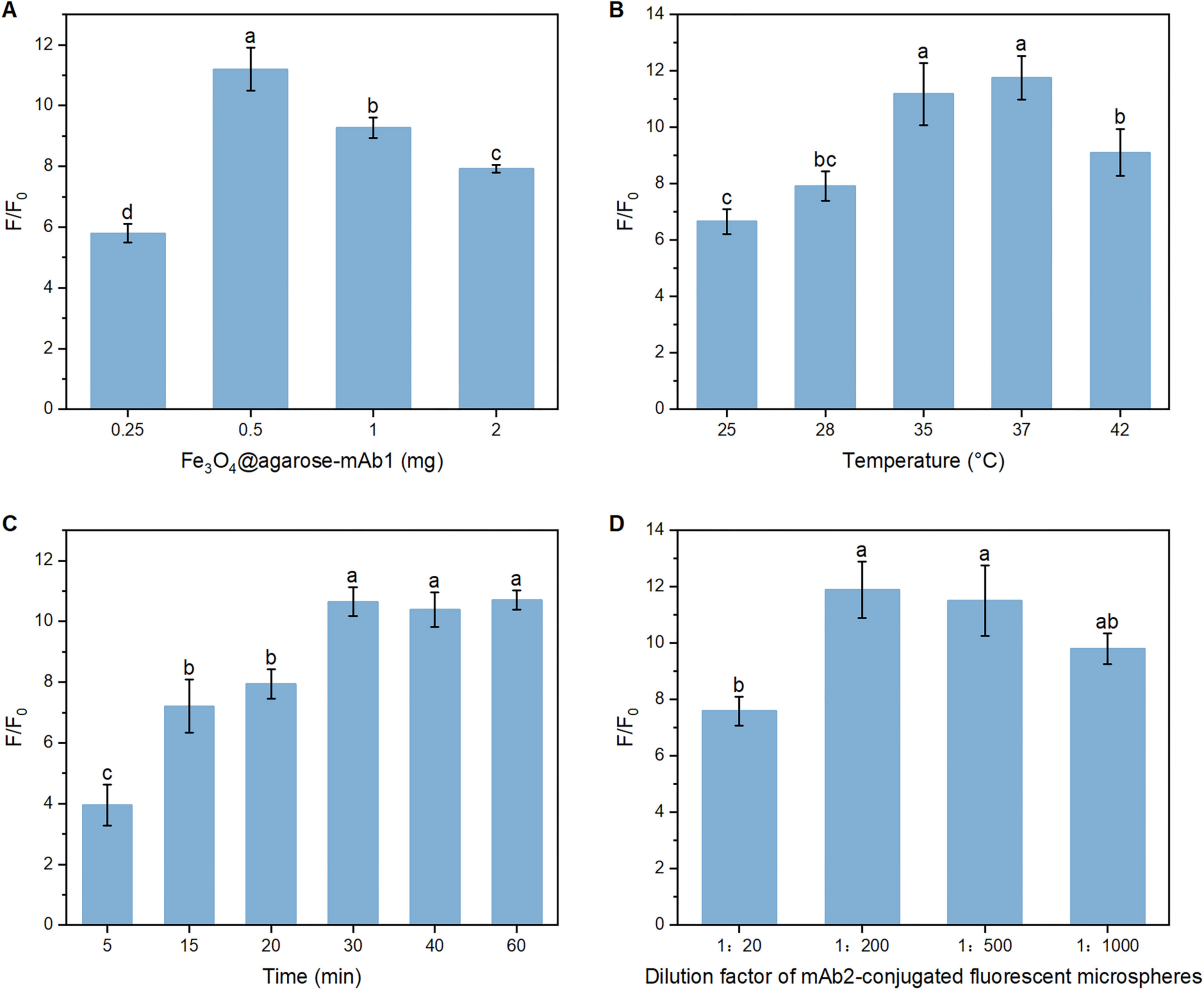
Signal-to-noise ratio of fluorescence intensity under varying conditions of **(A)** magnetic microspheres dosage, **(B)** reaction temperature, **(C)** incubation time, and **(D)** dilution ratio of mAb2-conjugated fluorescent microspheres. Error bars indicate the standard deviation (SD) of the mean calculated from three replicates. One-way ANOVA with Tukey’s *post-hoc* test was used for comparisons among groups. Bars with different letters indicate significant differences at *p* < 0.05.

Using single-factor optimization, several parameters were systematically optimized to achieve the best analytical performance, including magnetic microspheres dosage, reaction temperature, incubation time, and the dilution ratio of mAb2-conjugated fluorescent microspheres. A fixed concentration of 12.5 ng/mL KPC protein was used in all optimization experiments, with PBS serving as the negative control to observe background signals. The fluorescence intensity measured from the KPC sample was defined as *F*, whereas that measured from the blank PBS control was defined as *F*_0_. The signal-to-noise ratio was calculated as *F*/*F*_0_. One-way ANOVA was performed, followed by Tukey’s *post-hoc* test for intergroup difference analysis. As shown in Figure 4A, with the increase in magnetic microspheres dosage, the amount of captured KPC protein increased accordingly, resulting in a stronger measured fluorescence signal. Meanwhile, the background signal also showed an upward trend. When the magnetic microspheres dosage exceeded 0.5 mg, the signal-to-noise ratio gradually decreased (Figure 5A). ANOVA revealed statistical differences among all groups. Therefore, the optimal magnetic microspheres dosage was determined to be 0.5 mg. As shown in Figure 4B, the fluorescence signal gradually increased as the reaction temperature rose from 25 to 37°C. At 37°C, both the fluorescence signal and its difference from the background signal reached the maximum values. When the temperature rose to 42°C, the signal decreased. ANOVA revealed no statistical difference in signal-to-noise ratio between 35 and 37°C (Figure 5B). To standardize subsequent experimental conditions, 37°C, which yielded the highest average total fluorescence intensity, was selected as the optimal reaction temperature. Both incubation periods were set to the same duration. As shown in Figure 4C, the fluorescence signal gradually increased when the incubation time ranged from 5 to 30 min. After 30 min, the fluorescence intensity tended to stabilize. ANOVA revealed no statistical differences in signal-to-noise ratio among the 30 min, 40 min and 60 min groups (Figure 5C). These results indicated that the reaction between KPC protein and the antibodies basically reached saturation at approximately 30 min. To achieve the goal of rapid detection, 30 min was selected as the optimal incubation time. As shown in Figures 4D, 5D, the fluorescence intensity decreased with increasing dilution ratio of the mAb2-conjugated fluorescent microspheres, while the signal-to-noise ratio initially increased and then decreased. ANOVA revealed statistical differences between the 1:200 and 1:20 groups. Therefore, a dilution ratio of 1:200 with both relatively high fluorescence intensity and a high signal-to-noise ratio was selected as the optimal ratio for the mAb2-conjugated fluorescent microspheres. The optimal detection conditions were ultimately determined as follows: magnetic microspheres dosage of 0.5 mg, reaction temperature of 37°C, incubation time of 30 min, and mAb2-conjugated fluorescent microspheres dilution ratio of 1:200. Insufficient signal was observed with shorter incubation times or lower temperatures, whereas excessive magnetic microspheres or improper antibody dilution reduced detection performance.

### 3.3 Analytical performance

**FIGURE 6:**
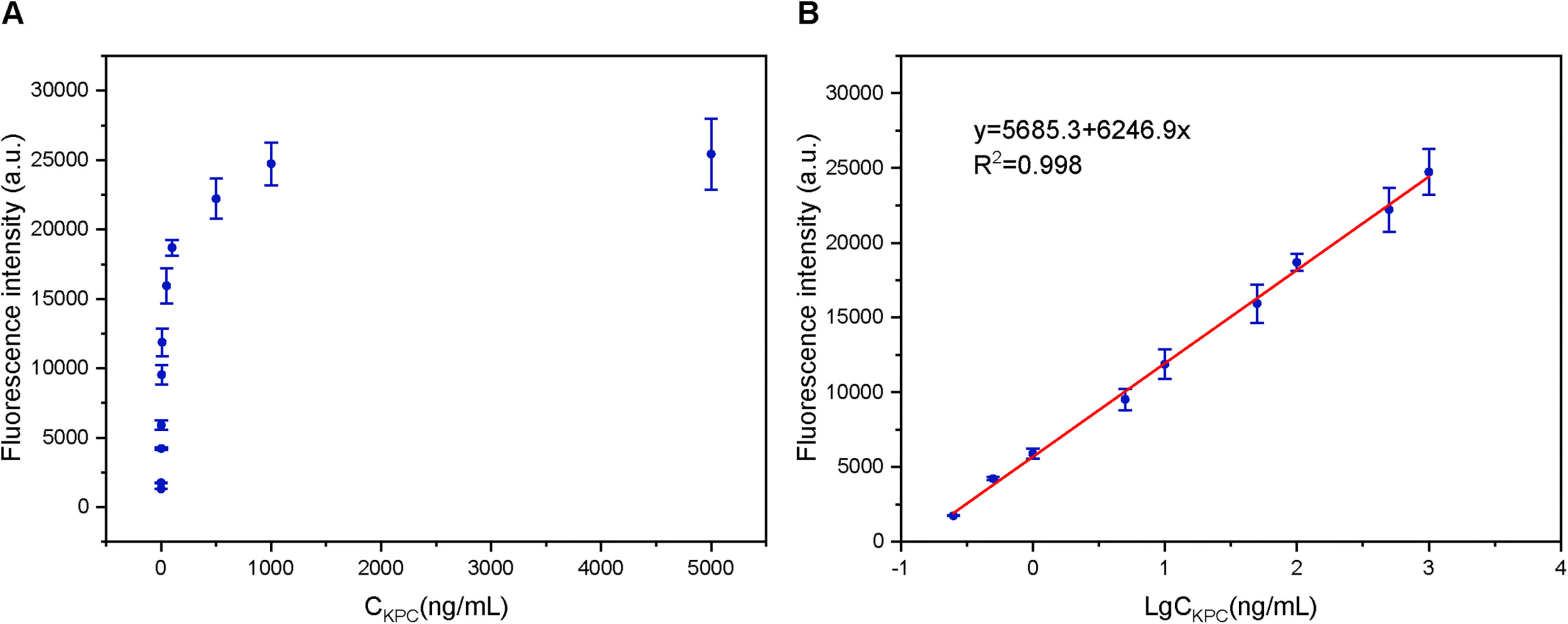
(A) Fluorescence response to varying KPC protein concentrations. **(B)** Linear relationship between fluorescence intensity and the logarithm of KPC concentration (linear range: 0.25–1,000 ng/mL). Error bars indicate the standard deviation of the mean from three replicates.

To establish the linear range and determine the sensitivity of the method, a series of standard KPC protein solutions with concentrations of 0.125, 0.25, 0.5, 1, 5, 10, 50, 100, 500, 1,000, and 5,000 ng/mL were analyzed under the optimal detection conditions. Figure 6A illustrates that the fluorescence intensity increased with increasing KPC concentration. As presented in Figure 6B, a good linear relationship was observed between the fluorescence signal intensity and the logarithm of KPC concentration in the range of 0.25–1,000 ng/mL. The regression equation was determined as *F* = 5685.3 + 6246.9 lg*C* with a correlation coefficient (R^2^) of 0.998, where *F* represents the fluorescence intensity and *C* represents the KPC protein concentration. The LOD was estimated to be 0.194 ng/mL based on a signal-to-noise ratio (S/N) of 3. Specifically, 10 independent blank samples were measured, and the average detection signal plus three times its standard deviation was substituted into the calibration curve equation to calculate the LOD. Similarly, the limit of quantification (LOQ) was determined to be 0.228 ng/mL using an S/N ratio of 10. The relative standard deviations (RSDs) of the nine detection concentrations within the linear range ranged from 1.4 to 8.3%, indicating that the method exhibits excellent repeatability.

**FIGURE 7:**
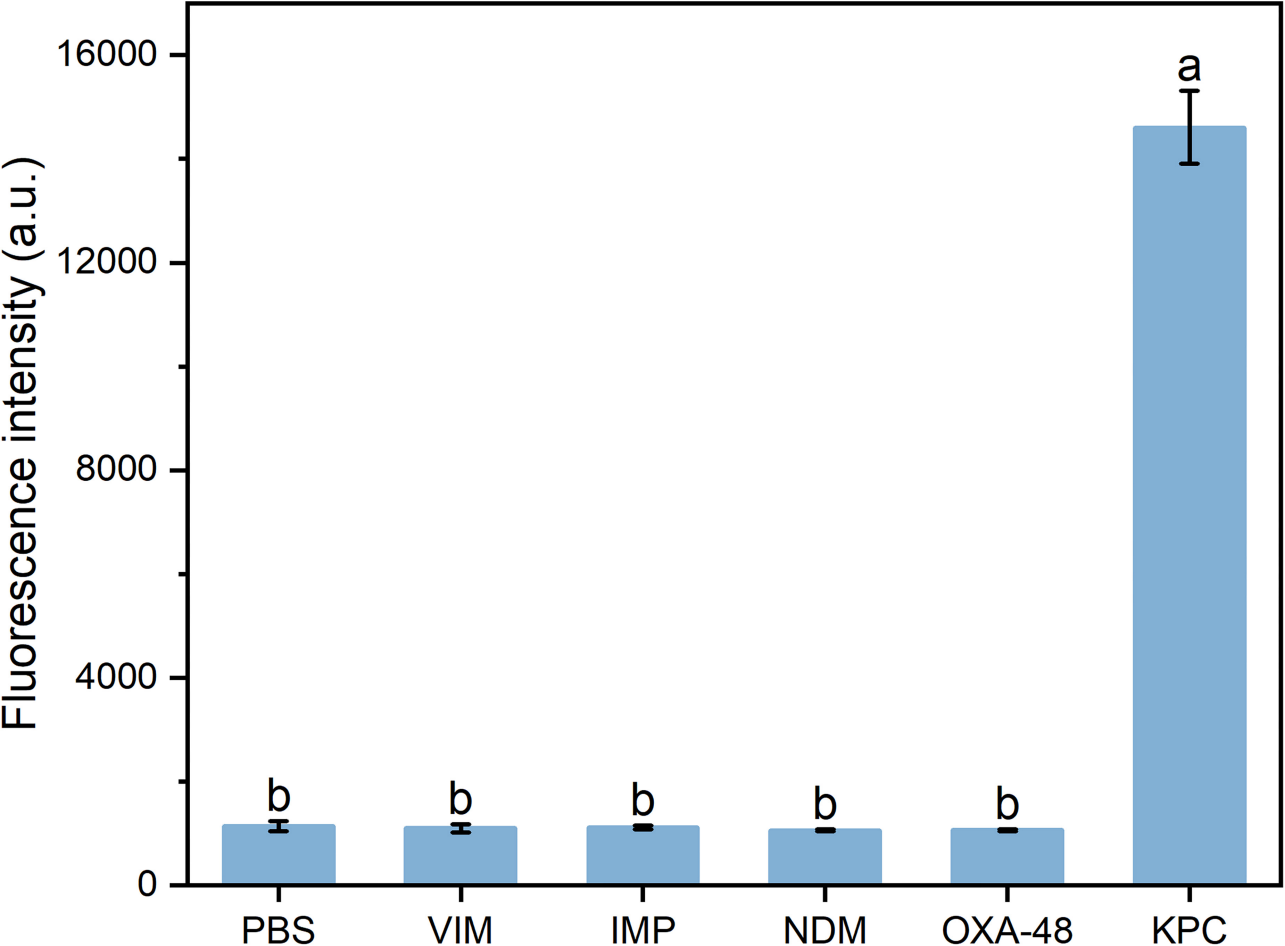
Evaluation of the specificity of fluorescence immunoassay using different enzyme proteins. Error bars represent the standard deviation (SD) of the calculated mean value. One-way ANOVA with Tukey’s *post-hoc* test was used for comparisons among groups. Bars with different letters indicate significant differences at *p* < 0.05.

**FIGURE 8:**
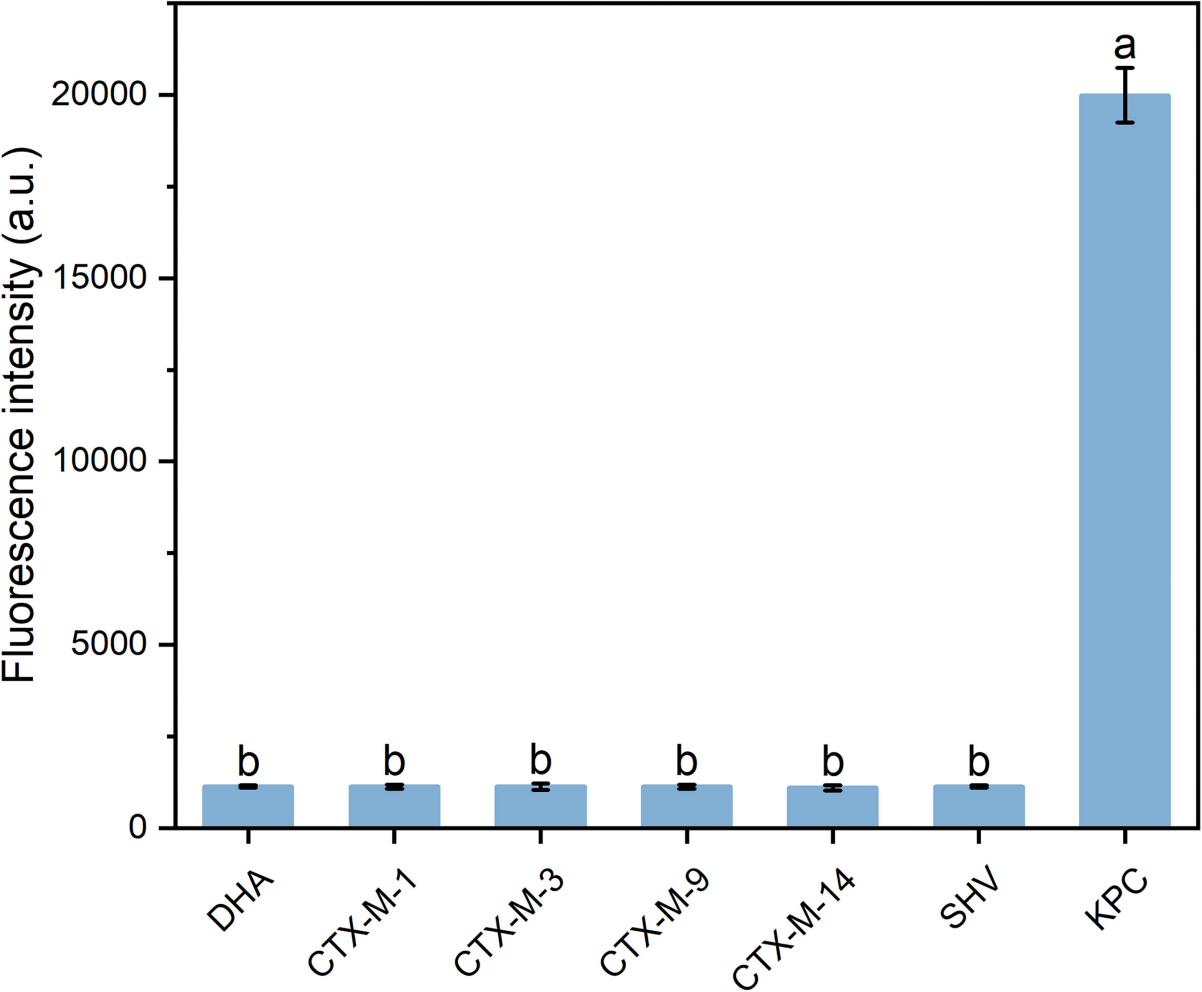
Evaluation of the specificity of fluorescent immunoassay using different clinical isolates. Error bars represent the standard deviation (SD) of the calculated mean value. One-way ANOVA with Tukey’s *post-hoc* test was used for comparisons among groups. Bars with different letters indicate significant differences at *p* < 0.05.

To evaluate the specificity of the method, PBS was used as a negative control, while KPC, VIM, IMP, NDM, and OXA-48 proteins were tested at identical concentrations of 25 ng/mL. Only the KPC protein elicited a significant fluorescence response, whereas the other carbapenemases at the same concentration elicited negligible signals, all of which were below the LOD of the method (Figure 7). No statistical differences in fluorescence intensity were observed between these carbapenemase groups and the negative control. However, the KPC group showed a significant difference when compared to each of the other carbapenemase groups and the negative control. Subsequently, the interference of other β-lactamases on KPC detection was validated using clinically isolated strains. As shown in Figure 8, all non-KPC-producing strains exhibited fluorescence signals below the detection limit of the assay. In contrast, the KPC-producing group had significantly higher fluorescence intensity, with a significant difference from each non-KPC group. These findings underscore that the developed method exhibits high specificity toward KPC protein, reliably distinguishing it from other common carbapenemases and β-lactamases and thereby ensuring the accurate detection of KPC.

To evaluate the accuracy of the developed method and investigate the potential influence of bacterial matrices on detection, spiked recovery experiments were performed. Lysates of *Klebsiella pneumoniae* ATCC BAA-1706 and *Pseudomonas aeruginosa* ATCC 27853 that were previously verified by PCR to not carry *bla*_KPC_ were selected as the matrices. These lysates were spiked with KPC protein at three concentration levels and analyzed by the present method. The recovery rates of KPC protein in the two bacterial strain matrices ranged from 96.7 to 108.4% (Table 1). These results demonstrate the good accuracy of the method and its low susceptibility to bacterial matrix interference, thus fulfilling the requirements for the accurate quantitative analysis of real samples.

**TABLE 1:** Results of detecting KPC in bacterial lysates samples by the present method (*n* = 3).

Samples	Spiked (ng/mL)	Found (ng/mL)	Recovery (%)	RSD (%)
*Klebsiella pneumoniae* ATCC BAA-1706	1.25	1.31	104.6	6.2
	25	27.10	108.4	3.2
	125	120.93	96.7	4.2
*Pseudomonas aeruginosa* ATCC 27853	1.25	1.34	106.9	4.8
	25	25.01	100.0	3.4
	125	131.86	105.5	1.1

### 3.4 Method Comparison

#### 3.4.1 Comparison with PCR

Both the proposed method and PCR results are shown in Table 2. Among the 31 clinical isolates, the proposed method identified 16 KPC-producing strains and 15 non-KPC-producing strains, which were completely consistent with the PCR results (Kappa = 1). The concordance rate between this method and the PCR method was 100%.

**TABLE 2:** Concordance of KPC detection results between PCR and the proposed method for clinical isolates.

Methods		PCR		Total	Kappa
		Positive	Negative		
This proposed method	Positive	16	0	16	1
	Negative	0	15	15	
	Total	16	15	31	

#### 3.4.2 Comparison with CGIA

**FIGURE 9:**
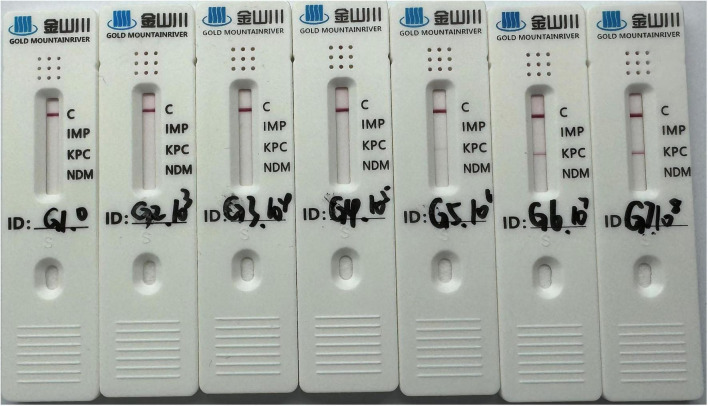
Results of the CGIA for samples at different bacterial concentrations, recorded at 10 min.

The detection results of the commercial CGIA are illustrated in Figure 9: samples at 10^3^–10^5^ CFU/mL tested negative, those at 10^6^ CFU/mL showed weak positive signals, while samples at 10^7^ and 10^8^ CFU/mL displayed clear positive results. The results of the proposed method are detailed in Table 3. At 10^4^ CFU/mL, the detected signals fell between the LOD and LOQ. And samples containing 10^5^ CFU/mL produced signals exceeding the LOQ, within the method’s established linear range. Additionally, according to the reagent instructions, the LOD of the commercial CGIA is 0.5 ng/mL, which is higher than that of the proposed method (0.194 ng/mL). The comparative results demonstrate that the proposed method achieves a lower detection limit than the commercial CGIA, suggesting its potential utility for detecting lower concentrations or low-expression bacterial samples.

**TABLE 3:** Performance comparison between the present method and CGIA (*n* = 3).

Bacterial concentration (CFU/mL)	Fluorescence intensity (a.u.)	Result by the present method	Result by CGIA
0	1020.9	Negative	Negative
10^3^	1193.0	Negative	Negative
10^4^	1251.5	>LOD	Negative
10^5^	3053.8	>LOQ	Negative
10^6^	9526.8	>LOQ	Weakly positive
10^7^	14579.3	>LOQ	Positive
10^8^	20713.1	>LOQ	Positive

## 4 Discussion

CRGNB infections are prevalent in multiple regions worldwide and are associated with high morbidity and mortality rates, particularly in low- and middle-income countries. Inadequate or inappropriate treatment of these infections can further aggravate the phenomenon of antimicrobial resistance (Pierce et al., 2020). Over the past decade, numerous novel antibacterial agents targeting critical pathogens like CRE have been developed and approved for clinical use (Lasko and Nicolau, 2020). However, the emergence of resistance has been consistently observed following the introduction of these new drugs into clinical practice (Boattini et al., 2024). Furthermore, a study demonstrated that imipenem increased mortality in mice infected with MDR *Klebsiella pneumoniae* and suggested that inappropriate antibiotic use in the context of MDR bacterial infections, may lead to destructive inflammatory responses (Ye et al., 2021). This reality underscores the urgency and importance of persistently investigating resistance mechanisms, the prudent and rational use of existing antimicrobial agents, and actively developing novel therapeutic strategies. From a clinical perspective, in this dynamic struggle between humans and bacteria, rational drug utilization serves as the critical link, while rapid and precise detection methods constitute an indispensable tool.

The type of carbapenemase produced by resistant strains directly shapes their susceptibility patterns to the latest β-lactam/β-lactamase inhibitor combinations, thereby determining the selection of optimal treatment regimens and ultimately affecting patient survival. For instance, KPC-producing strains respond well to ceftazidime-avibactam, meropenem-vaborbactam, and imipenem-relebactam, whereas metallo-β-lactamases (NDM, VIM, IMP) are not inhibited by these agents and call for completely distinct therapeutic strategies such as aztreonam-avibactam or cefiderocol (Satlin et al., 2022). This further highlights that precise carbapenemase types identification is essential for delivering individualized, evidence-based antimicrobial treatment and achieving favorable clinical outcomes.

This study established a magnetic separation-based fluorescent immunoassay for highly sensitive and quantitative detection of KPC. The assay can be completed within approximately 1 h, achieving a detection limit of 0.194 ng/mL, and exhibits no cross-reactivity with four other common carbapenemase types (VIM, IMP, NDM, and OXA-48). This method employs Fe_3_O_4_@agarose-IDA as the solid-phase carrier for KPC capture, which has a large specific surface area that can immobilize a higher density of antibodies. When incubated with agitation, the microspheres form a uniform suspension to enhance the contact area with target analytes. While efficiently enriching KPC, it also reduces both sample volume requirements and reaction time. Fluorescent probes prepared by time-resolved fluorescent microspheres have the advantages of low background interference and high signal loading capacity compared with conventional luminescent materials, and can achieve effective signal amplification.

This technology offers the advantages of a low detection limit and a wide linear range, providing a promising solution to address the challenge of early clinical diagnosis of KPC-producing CRGNB. Researchers have reported that the bacterial concentration in positive blood cultures ranges from 2 × 10^7^ to 7 × 10^9^ CFU/mL (median, 5 × 10^8^ CFU/mL) (Christner et al., 2010). The sensitivity of our assay is likely to be well below this range, suggesting its potential for direct detection of KPC enzymes in blood samples from patients with severe bloodstream infections or other specimens with high bacterial loads, such as pus or drainage fluids. This capability could reduce or eliminate culture time, thereby significantly shortening the total diagnostic turnaround time.

Furthermore, this study has achieved quantitative detection of KPC. Current phenotypic and genotypic methods used in clinical practice primarily provide qualitative results. However, KPC expression levels can be influenced by complex factors such as bacterial physiological status, gene copy number, and regulatory mechanisms. Not all KPC-producing strains exhibit the same level of resistance phenotype. The ability to quantitatively measure the KPC concentration establishes a foundation for future exploration of correlations between enzyme expression, minimum inhibitory concentration (MIC) values, and clinical treatment outcomes. This advancement holds promise for enabling more profound resistance profiling and facilitating precision therapy. Recent studies have revealed that CRKP can secrete outer membrane vesicles (OMVs) carrying KPC enzymes, which hydrolyze antibiotics in a dose- and time-dependent manner to protect *Pseudomonas aeruginosa* in polymicrobial infections from imipenem-mediated killing. These OMVs can also induce the emergence of a carbapenem-resistant subpopulation in *Pseudomonas aeruginosa* (Zhang et al., 2023). This finding suggests that the quantitative detection of KPC can provide additional insights into the dynamics of antimicrobial resistance and related mechanistic investigations in mixed infections.

Compared with PCR, the proposed method reduces the requirements for equipment and operator expertise while ensuring detection accuracy. Relative to mCIM and Carba NP tests, it offers shorter turnaround time and enables precise carbapenemase typing. Notably, compared with immunochromatographic assays that are also based on immunological principles, the major advantages of the present method lie in its high sensitivity and full quantitative capability. These two approaches are complementary and can be adopted by laboratories at different levels according to practical conditions. Immunochromatographic strips undoubtedly offer superior simplicity of operation, making them suitable for rapid point-of-care screening and use by non-specialized personnel. In contrast, the additional equipment required for this method includes an oscillating mixer, a spectrofluorophotometer, and a magnet or magnetic separation rack, all of which can be readily met by medium-sized and above laboratories. As revealed by the above comparisons, this method is particularly suitable for microbiology laboratories in regional hospitals or teaching hospitals. On the one hand, it can provide critical references for the diagnosis and treatment of critically ill patients. On the other hand, it can also serve as a research tool for antimicrobial resistance surveillance and analysis.

This study also has certain limitations. Firstly, like other existing technologies, this method detects total KPC protein in bacterial lysates and cannot directly identify the bacterial species producing the enzyme. Thus, additional bacterial identification is required to meet clinical needs. Secondly, the current work is in the proof-of-concept and performance evaluation phase, and the number of strains used for validation was limited. Future research should validate the performance of this method using a larger and more diverse collection of strains, including a broader range of β-lactamase-producing strains and strains from different clinical sources, to further enhance the generalizability and practical applicability of this method. The excellent analytical performance demonstrated here needs to be further validated for diagnostic efficacy in prospective, large-sample clinical cohort studies. Moreover, the detection coverage of rare KPC variants that have emerged continuously in recent years requires ongoing assessment and updates. At present, this method has only been validated using pure bacterial cultures. Whether it can maintain the same sensitivity and specificity in complex clinical sample matrices, such as sputum, blood, or drainage fluid, requires further optimization and verification. If this method can be applied to direct clinical samples, it would be feasible to perform simultaneous bacterial identification and carbapenemase resistance detection on specimens from critically ill infected patients based on clinical practice, thereby achieving the goal of rapidly guiding antimicrobial therapy and offering a novel possibility for future anti-infective treatment strategies. Finally, to comprehensively address clinical needs, it is necessary to continue developing the detection of enzyme types other than KPC and the combined detection of multiple enzyme types. This represents a critical direction for our ongoing research.

Despite these limitations, the fluorescent immunoassay developed in this study undoubtedly offers a new pathway for the rapid detection of KPC. The quantitative and highly sensitive characteristics of this assay make it not only a diagnostic tool but also a potentially valuable research instrument. It can be used for monitoring the expression kinetics of KPC under antibiotic pressure or evaluating the efficacy of novel enzyme inhibitors. Ultimately, advancing the clinical application of such rapid diagnostic technologies will directly contribute to the implementation of precise antibiotic stewardship strategies, optimize the use of new therapeutic agents, thereby improving patient outcomes and curbing the further spread of antimicrobial resistance.

## 5 Conclusion

This study successfully developed a rapid and sensitive sandwich immunoassay based on magnetic microparticles and fluorescently labeled antibodies for the quantitative detection of KPC enzymes produced by bacteria. The assay requires only approximately 1 h of experimental time and demonstrates excellent sensitivity, with a detection limit of 0.194 ng/mL. It effectively identifies bacterial specimens with low bacterial loads or low enzyme expression levels, thereby potentially reducing the risk of clinical false negatives. The assay exhibits a good linear relationship between the fluorescent signal and KPC concentration within the clinically relevant range of 0.25–1,000 ng/mL. This method represents the first realization of quantitative analysis for KPC carbapenemase, enabling investigation into the correlation between bacterial resistance levels and enzyme expression. This capability extends beyond the information dimension provided by traditional qualitative or semi-quantitative detection methods. As a promising complementary diagnostic tool, this assay holds great potential for application in clinical microbiology laboratories for the early and rapid identification of KPC-producing strains and the assessment of antimicrobial resistance. Future studies will focus on expanding clinical sample validation by testing the method’s performance on direct clinical specimens, such as blood, urine, and cerebrospinal fluid. Additionally, efforts will extend to detecting other carbapenemase types, developing multiplex detection capabilities, and establishing automated platforms.

## Data Availability

The original contributions presented in the study are included in the article/Supplementary material, further inquiries can be directed to the corresponding authors.
