# Supplementary material for: Rapid and high-sensitivity quantitative fluorescent immunoassay for detection of Klebsiella pneumoniae carbapenemase
Source: Front Microbiol. 2026 Apr 29;17:1806227. doi: 10.3389/fmicb.2026.1806227 (PMC13170100; doi:10.3389/fmicb.2026.1806227)
Supplement: Supplementary file 1 [file Table_1.docx]

**Supplementary Information**

**Rapid and High-Sensitivity Quantitative Fluorescent Immunoassay for Detection of *Klebsiella Pneumoniae*** **Carbapenemase**

Menglu Mao^1†^, Keke Li^2†^, Chenyao Lin^1^, Xuedan Qiu^1^, Zhen Zhang^4^, Dumei Ma^3*^, Qingcao Li^1,5*^

^1^Department of Clinical Laboratory, The Affiliated Lihuili Hospital of Ningbo University, Ningbo, 315000, China

^2^Genobio Pharmaceutical Co., Ltd., Tianjin, 300480, China

^3^Key Laboratory of Advanced Mass Spectrometry and Molecular Analysis of Zhejiang Province, School of Materials Science and Chemical Engineering, Institute of Mass Spectrometry, Ningbo University, Ningbo, 315211, China

^4^School of Laboratory Medicine and Bioengineering, Hangzhou Medical College, Hangzhou, 310053, China

^5^State Key Laboratory for Diagnosis and Treatment of Severe Zoonostic Infectious Disease, Wuhan, 430210, China

†These authors contributed equally to this work and share first authorship

* Correspondence:

Dumei Ma, Qingcao Li

Email: madumei@nbu.edu.cn (Dumei Ma); lqc_lab@163.com (Qingcao Li)

**Synthesis of Fe_3_O_4_@agarose-IDA**

Firstly, magnetic agarose microsphere cores were fabricated by a water-in-oil emulsion method. Briefly, the aqueous phase was prepared by mixing 0.1 g of Fe_3_O_4_ nanoparticles with 10 mL of a 4% agarose solution at 95 °C under mechanical stirring at 1000 rpm; the oil phase was prepared by mixing 75 mL of liquid paraffin with 2.8 mL of Span 80 at 80 °C. The aqueous phase was then rapidly transferred into the oil phase, and then subjected to high-speed shear emulsification at 20,000 rpm for 15 minutes. The mixture was then transferred to an ice bath, and emulsification was continued for 10 minutes. Fe_3_O_4_@agarose microspheres were subsequently collected by magnetic separation and washed thoroughly with petroleum ether, ethanol, and deionized water. Secondly, 0.1 g of Fe_3_O_4_@agarose microspheres was activated by mixing with 12 mL of NaOH (1 mol/L) and 0.5 mL of NaBH_4_ (0.5 mg/mL), followed by stirring at 25 °C and 400 rpm for 30 minutes. Then 6.0 mL of epichlorohydrin in 18 mL DMSO was added, and the reaction proceeded at 30 °C for 8 hours. Fe_3_O_4_@agarose-epoxy microspheres were obtained through magnetic separation and washed with deionized water. Finally, 0.1 g of Fe_3_O_4_@agarose-epoxy microspheres was added into an activation system composed of 0.55 g IDA, 15.5 mL of 1 mol/L Na_2_CO_3_ solution, and 0.88 mL of 0.5 mg/mL NaBH_4_ solution, followed by a shaking reaction at room temperature for 12 hours. After the reaction was completed, the resulting carboxylated magnetic microspheres (Fe_3_O_4_@agarose-IDA) were collected using a magnet and washed with deionized water.

Supplementary Table 1

Information of clinical isolates (*Klebsiella pneumoniae*) for specificity validation.

| Group | No. | Presence of other β-lactamase |
| --- | --- | --- |
| DHA | 1 | SHV |
|  | 2 | SHV |
|  | 3 | SHV |
| CTX-M-1 | 4 | SHV |
|  | 5 | SHV |
|  | 6 | SHV |
| CTX-M-3 | 7 | SHV |
|  | 8 | – |
|  | 9 | – |
| CTX-M-9 | 10 | CTX-M-14 + SHV |
|  | 11 | CTX-M-14 + SHV |
|  | 12 | – |
| CTX-M-14 | 13 | DHA + CTX-M-9 + SHV |
|  | 14 | CTX-M-9 + SHV |
|  | 15 | CTX-M-3 + SHV |
| SHV | 16 | – |
|  | 17 | – |
|  | 18 | – |
| KPC | 19 | SHV |
|  | 20 | SHV |
|  | 21 | SHV |

Supplementary Table 2

Information of clinical isolates used for methodological comparison.

|  | | Species | Type of carbapenemases | |
| --- | --- | --- | --- | --- |
| KPC-producing isolates (n = 12) | | | |  |
|  | *Klebsiella pneumoniae* (n = 11) | | | KPC-2 |
|  | *Pseudomonas aeruginosa* (n = 1) | | | KPC-2 |
| Double-carbapenemase producers (n = 4) | | | |  |
|  | *Klebsiella pneumoniae* (n = 2) | | | KPC-2 + IMP-1 |
|  | *Klebsiella pneumoniae* (n = 2) | | | KPC-2 + NDM-1 |
| Other carbapenemases producers (n = 6) | | | |  |
|  | *Klebsiella pneumoniae* (n = 2) | | | IMP-1 |
|  | *Klebsiella pneumoniae* (n = 3) | | | NDM-1 |
|  | *Klebsiella pneumoniae* (n = 1) | | | OXA-48 |
| Non-carbapenemase-producing CRE (n = 2) | | | |  |
|  | *Klebsiella pneumoniae* (n = 2) | | | None |
| Carbapenem-susceptible isolates (n = 7) | | | |  |
|  | *Klebsiella pneumoniae* (n = 3) | | | None |
|  | *Pseudomonas aeruginosa* (n = 2) | | | None |
|  | *Escherichia coli* (n = 1) | | | None |
|  | *Acinetobacter baumannii* (n = 1) | | | None |
